# Detailed analysis of paternal knockout *Grb10* mice suggests effects on stability of social behavior, rather than social dominance

**DOI:** 10.1111/gbb.12571

**Published:** 2019-04-29

**Authors:** Kira D. A. Rienecker, Alexander T. Chavasse, Kim Moorwood, Andrew Ward, Anthony R. Isles

**Affiliations:** ^1^ MRC Centre for Neuropsychiatric Genetics and Genomics, Neuroscience and Mental Health Research Institute, School of Medicine Cardiff University Cardiff UK; ^2^ Department of Biology and Biochemistry University of Bath Bath UK

**Keywords:** age, barbering, genomic imprinting, social dominance, social isolation

## Abstract

Imprinted genes are highly expressed in monoaminergic regions of the midbrain and their functions in this area are thought to have an impact on mammalian social behaviors. One such imprinted gene is *Grb10*, of which the paternal allele is generally recognized as mediating social dominance behavior. However, there has been no detailed study of social dominance in *Grb10* ^*+/p*^ mice. Moreover, the original study examined tube‐test behavior in isolated mice 10 months of age. Isolation testing favors more territorial and aggressive behaviors, and does not address social dominance strategies employed in group housing contexts. Furthermore, isolation stress impacts midbrain function and dominance related behavior, often through alterations in monoaminergic signaling. Thus, we undertook a systematic study of *Grb10* ^*+/p*^ social rank and dominance behavior within the cage group, using a number of convergent behavioral tests. We examined both male and female mice to account for sex differences and tested cohorts aged 2, 6 and 10 months to examine any developments related to age. We found group‐housed *Grb10* ^*+/p*^ mice do not show evidence of enhanced social dominance, but cages containing *Grb10* ^*+/p*^ and wild‐type mice lacked the normal correlation between three different measures of social rank. Moreover, a separate study indicated isolation stress induced inconsistent changes in tube test behavior. Taken together, these data suggest future research on *Grb10* ^*+/p*^ mice should focus on the stability of social behaviors, rather than dominance per se.

## Graphical abstract

Mice lacking paternal expression of imprinted Grb10 do not show increased social dominance behaviour relative to wild‐type littermates. However, following a period of social isolation a sexually‐dimorphic social dominance phenotype does emerge.

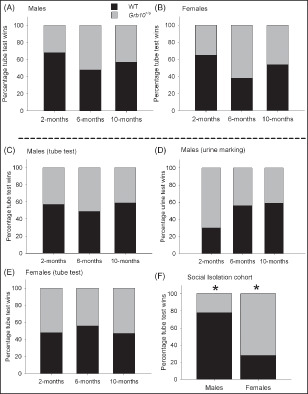

## 1 INTRODUCTION

Imprinted genes are defined by their monoallelic, parent‐of‐origin dependent expression originating from differential epigenetic marks established in the germline.^1^ This class of genes is highly expressed in the central nervous system and significantly impacts brain development and adult behaviors.^2^ The paternally expressed copy of the imprinted gene *Grb10* (growth factor receptor bound protein 10) is expressed in the developing and adult brain, and we have previously established a potential link to social dominance in mice with disruption of the paternally inherited allele (*Grb10* ^+/p^).^3^ Murine *Grb10* is located on proximal chromosome 11 and encodes a cellular adapter protein belonging to the small Grb7/Grb10/Grb14 family.^4^, ^5^ This protein has an inhibitory effect on signaling through receptor tyrosine kinases, including the insulin receptor and insulin‐like growth factor receptor.^6^ Paternal *Grb10* is highly expressed in the midbrain and hindbrain, including regions such as the ventral tegmental area, the substantia nigra pars compacta, the dorsal raphe nucleus, thalamus and hypothalamus, and is neuron‐specific.^3^, ^7^

Male *Grb10* ^*+/p*^ mice 10 months of age were previously reported to be significantly less likely to back down in the Lindzey tube test. This correlated with an elevated incidence of facial barbering in cages containing *Grb10* ^*+/p*^ mutants.^3^ Both measures are considered indicators of social dominance.^8^, ^9^, ^10^ However, in the original study tube testing was not conducted within an animal's normal cage group, and also took place after mice were isolated for an extended period to determine whether the barbering was self‐inflicted.^3^ Social isolation impacts midbrain function and dominance‐related behaviors, often through alterations in monoaminergic signaling.^11^, ^12^ In periods of isolation between 14 and 28 days, this includes alterations in tyrosine hydroxylase transcription, and over 3 months this includes changes in epigenetic marks and writer/eraser activity in the midbrain.^11^, ^13^ Even short periods alter signaling and connectivity. Acute social isolation over 24 hours potentiates synapses onto dopamine neurons in the dorsal raphe nucleus (DRN) and alters their glutamate receptor composition.^14^ Furthermore, social rank itself impacts the subjective experience of isolation, as dominant mice are more sensitive to the behavioral effects of manipulating DRN dopaminergic activity through optogenetic activation and inhibition.^14^

Here we systematically explore social dominance behavior of *Grb10* ^*+/p*^ mice. We used convergent measures to assess dominance behavior in socially housed *Grb10* ^*+/p*^ mice, including the stranger‐ and social‐encounter Lindzey tube tests, the urine marking test, and characterization of barbering behavior. Both male and female cohorts were used to test for any sex differences. Also, cohorts at 2, 6 and 10 months of age were tested in a cross‐sectional study designed to account for any differences that may develop with age. Given the extensive changes to midbrain synaptic function, monoaminergic signaling and epigenetic regulation induced by social isolation, we saw a need to determine whether the isolation period from the earlier experiment^3^ impacted the tube test phenotype observed in *Grb10* ^*+/p*^ mice. We therefore replicated the dominance testing of isolated *Grb10* ^*+/p*^ mice 10 months of age to determine whether isolation stress was required to precipitate the phenotype. Our results indicate *Grb10* ^*+/p*^ mice are not more dominant, but may show a social instability phenotype.

## 2 MATERIALS AND METHODS

### 2.1 Animals

All procedures were conducted in accordance with the requirements of the UK Animals (Scientific Procedures) Act 1986, under the remit of Home office license number 30/3375 with ethical approval at Cardiff University. *Grb10* heterozygous knockout mice on a B6CBAF1/J background were previously created as described in Garfield et al^3^ using a LacZ:neomycin gene‐trap cassette interrupting exon 7.^3^, ^7^ This mouse colony was derived via embryo transfer from a colony in Bath and maintained on exactly the same mixed genetic background. Specifically, breeding stock was maintained with either a B6CBA F1/crl line from Charles River or with an in house mixed B6CBA F1/crl × B6CBA F1/J background. Experimental animals were generated by crossing wild‐type (WT) breeding stock with the desired parent of origin heterozygous *Grb10* ^+/−^ animal. Dams were placed in individual housing the week prior to full term. This measure was necessary to aid pre‐weaning ear clip identification and genotyping of the behavioral cohorts. Mice were weaned between P19 and P28 and sorted into genotype‐balanced social cages of 4 mice: 2 WTs, 2 *Grb10* ^*+/p*^ for behavioral testing. Male mice were genotyped prior to weaning to enable the cage set‐up. Females were weaned prior to genotyping and re‐sorted into the appropriate set‐up as soon as possible. Where possible, animals of the same birth litter were kept together.

All mice were housed in single‐sex, environmentally enriched cages (cardboard tubes, shred‐mats, chew sticks) of 1‐5 adult mice per cage (except for isolation study detailed below). Cages were kept in a temperature and humidity controlled animal holding room (21 ± 2°C and 50 ± 10% respectively) on a 12‐hour light‐dark cycle (lights on at 7:00 hours, lights off at 19:00 hours). All subjects had ad libitum access to standard rodent laboratory chow and water. Cages were cleaned and changed once a week at a regular time and day of the week for minimal disruption. Cages were not cleaned during multiple day testing of the same dominance test, and were half‐cleaned between tube testing and urine marking blocks.

### 2.2 Behavioral testing

**Figure 1:**
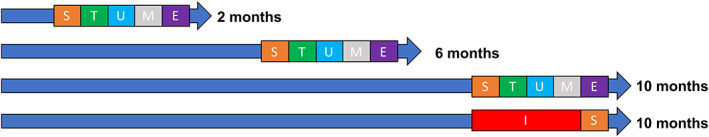
Experimental design. Four cohorts with both males and females (2, 6, 10 months and Isolation cohort at 10 months) underwent behavioral testing. Testing was limited to a 4‐week period and ended at the age indicated in the cohort name. The order of experiments was stranger encounter tube test (S), social encounter tube test (T), urine marking test (U; males only), marble burying test (M; not described in this paper) and elevated plus maze (E). The isolation cohort underwent a 30‐day isolation protocol (I) prior to the stranger encounter tube test (S)

The 2, 6 and 10‐month cohorts (but not the isolation cohorts) underwent dominance testing, in order, for: stranger tube test, social tube test and (males only) urine marking (Figure 1). Behavioral testing was limited to a 4‐week window to prevent age overlap with the other cohorts. Mice were handled as little as possible up until 1 week prior to the start of behavioral testing; then they were handled daily for 5 days before beginning testing. Testing was performed in a quiet room lit by a single indirect lamp bulb between 25 and 60 W. Match and cage numbers included in analysis for each behavioral test are reported in Tables 1, 2 and 3 below. A “match” constitutes a *Grb10* ^*+/p*^ vs WT encounter.

**Table 1:** Male matches—*Grb10* ^+/p^ vs WT

Age	Stranger tube matches	Social tube matches	Urine marking matches	Social isolation matches
2 months	28	56	44	—
6 months	23	51	52	—
10 months	23	46	46	10

*Note*: Matches between male *Grb10* ^*+/p*^ and WT mice included in analysis of social dominance testing.

**Table 2:** Female matches*—Grb10* ^+/p^ vs WT

Age	Stranger tube matches	Social tube matches	Social isolation matches
2 months	20	40	—
6 months	21	48	—
10 months	13	32	15

*Note*: Matches between female *Grb10* ^*+/p*^ and WT mice included in analysis of social dominance testing.

**Table 3:** Cage totals in hierarchy testing

Age	Male cages (social tube)	Male cages (urine marking)	Female cages (social tube)
2 months	15	11	10
6 months	13	13	12
10 months	12	12	8

*Note*: Cages of mice in each cohort (male and female) participating in hierarchy testing.

#### 2.2.1 Tube testing

The Lindzey tube test is an accepted measure of social dominance in mice and can be used to match subjects against strangers or cage‐mates.^8^ The stranger encounter and social encounter tube tests were conducted under identical conditions. For the stranger test, unfamiliar opponents were chosen from different home cages and different litters. Any socially housed WT opponents were housed in genotype‐balanced (2 WT, 2 *Grb10* ^*+/p*^) cages. Opponent mice were simultaneously presented to either end of a Perspex tube (30.5 m × 3.5 cm or 30 cm × 2.5 cm depending on weight class). Opponents met in the middle of the tube and outcome was scored when one animal was forced to back out. Losers were counted as the first animal with all four feet out of the tube. No time limit was imposed. Trials in which either opponent turned around in the tube, both mice backed out without confrontation, or both mice squeezed past each other were not counted (all instances of trial “failure”). In the stranger encounter tube test, animals were completely naïve to the test and mistrials were not re‐run (mistrials are listed in Supplementary information, Table S1). In the social encounter tube test, mistrials were re‐run on a separate day to complete the within‐cage hierarchy, but each opponent pair only underwent one successful trial. These paradigms were adopted to avoid any learning effects and to parallel testing procedures in reference 3. Each animal completed only one tube test per day. Testing was arranged to ensure genotype groups and individual mice underwent trials balanced by side of entry. In the stranger encounter tube test, opponents were weight matched to minimize differences across the whole cohort. To maximize trial numbers, no trials were eliminated based on weight. In approximately 77% of encounters, the heavier mouse was less than 15% heavier than the lighter mouse. There were no significant differences in body weight between *Grb10* ^*+/p*^ and WT mice in our colony across all three ages (2, 6 and 10 months) (See Supplementary Results).

#### 2.2.2 Urine marking

Mice were simultaneously placed in one compartment of a 30 × 30 × 30 cm box divided by a metal grid. A clear, smooth barrier was placed on top of the grid to prevent escape. Each compartment contained a 14 cm by 29.5 cm sheet of Whatman chromatography paper (3 mm, GE Healthcare UK Limited CAT No 3030‐2221). Each trial lasted 1 hour, at the end of which both mice were removed and the cages cleaned with 70% alcohol wipes. Marked paper was stained with Ninhydrin spray reagent (Sigma‐Aldrich N1286) and scored using a 1 cm^2^ grid overlay. All squares containing a scent mark were counted and used in a ratio against usable grid (total grid squares minus shredded sections and urine marks covering more than four consecutive squares). These scent marks/urine drops delineate territorial boundaries and contain chemical cues of social status.^15^ The winner of each encounter possessed the higher ratio of squares containing sent marks to usable grid.

#### 2.2.3 Barbering

The Dhalia Effect, or the whisker barbering effect, describes the tendency for the dominant mouse in the cage to trim the whiskers from subordinates, resulting in cages with just one unbarbered mouse.^9^ Barbering status was recorded at every behavioral testing session. Barbering was identified as the specific removal of whiskers (partial or complete); facial overgrooming could occur independently of barbering, and was thus noted, but not sufficient to confer a “barbered” status.

#### 2.2.4 Oestrus

Oestrus swabs were taken once per week following behavioral testing. Smears on gelatin‐coated slides were stained for 5 minutes using Cresyl fast violet and were identified under the microscope. On other days of testing, a visual assessment of oestrus status was recorded. Statistics pertaining to oestrus use the most closely associated oestrus stage and behavioral testing session.

#### 2.2.5 Isolation

Socially housed mice 9 months of age were placed in fresh individual housing for 30 days. Immediately following this isolation period, these mice, now 10 months of age, performed the stranger encounter tube test. Mice encountered one unfamiliar mouse of the opposite genotype (*Grb10* ^*+/p*^ or WT) per day for 3 days. Cage bedding was not changed during the testing period.

### 2.3 Statistics

Data analysis was performed using SPSS (versions 23 and 25). Data in diagrams are presented as mean ± SE of the mean, unless otherwise stated. Statistical significance underwent False Discovery Rate (FDR) corrections using the Benjamini‐Liu method.^16^, ^17^ FDR corrections were performed on all reported measures belonging to one task, and FDR corrections were separate between different tasks. FDR corrections were not carried out for groups of less than five statistical tests. The binomial test was conducted to determine if the proportion of *Grb10* ^*+/p*^ wins in “*Grb10* ^*+/p*^ *”* vs “wildtype” matches differed significantly from chance (0.5). Most individual mice were involved in two unique matches against cage mates of the opposite genotype. For example, “*Grb10* ^*+/p*^ A vs WT B” and “*Grb10* ^*+/p*^ A vs WT C” would be included in the analysis as independent matches. The related samples sign test and the Wilcoxon signed‐rank test were used to compare the difference in cage rank between the genotype groups. Hierarchies were established in each cage, with rank scored between 0 (least dominant) and 1 (most dominant), based on the number of wins divided by possible matches against cage mates. Data about differences and average genotype rank were presented as medians. The Mantel‐Haenszel test of trends was run to determine if there was a linear association between pairs of social tube test rank, urine marking rank and barbering rank in total male mice at each age cohort. For these statistical analyses, rank was described between 0 (0 wins against cage mates in the dominance tests) and 3 (three wins against cage mates in the dominance tests), or as 0 (barbered subordinate) and 1 (dominant barber).

## 3 RESULTS

### 3.1 Oestrus and Barbering status did not consistently predict tube test wins

Female mice are commonly excluded from social dominance assessments as they do not share some of the behaviors used to assess male social hierarchies, such as territorial marking and vocalizations to a potential mate. However, female mice can establish stable linear hierarchies in the Lindzey tube test. While test outcomes for male mice are strongly influenced by prior social experience, female mice primarily rely on intrinsic attributes to establish a hierarchy.^18^ Consequently, we tested both male and female mice. Before proceeding with analysis of our *Grb10* ^*+/p*^ vs WT matches, we analyzed stranger encounter tube tests in the female cohorts to determine whether we could predict tube test wins using oestrus status. In 16 social tube test matches pooled across the 2, 6 and 10‐month cohorts, a WT mouse judged to be in oestrus faced a WT mouse not in oestrus. A binomial test indicated the proportion of wins for WT females in oestrus (0.44) was not significantly different from chance (0.5), *P* = 0.804 (2‐tailed). Further analysis was performed on matches ignoring genotype. In 18 social tube test matches pooled across cohorts, a mouse judged to be in oestrus faced a mouse judged not to be in oestrus. In 9 matches, the mouse in oestrus was *Grb10* ^*+/p*^, and in the remaining 9 the mouse in oestrus was WT. A binomial test indicated the proportion of wins for mice in oestrus (0.33), regardless of genotype, was not significantly different from chance (0.5), *P* = 0.238 (2‐tailed). Based on these results, we justified ignoring oestrus stage in the statistical analysis of both stranger encounter and social encounter tube tests in the following sections.

We also analyzed the impact of barbering status on the stranger encounter tube test for males and females. In 16 matches in the 6‐month cohort, a barbered female mouse faced an unfamiliar, un‐barbered female mouse (of a different genotype, as per the design). In eight matches the barbered mouse was *Grb10* ^*+/p*^, and in the remainder, the barbered mouse was WT. A binomial test indicated the proportion of wins for barbered female mice 6 months of age (0.88) against unbarbered mice, regardless of genotype, was statistically different from chance (0.5), *P* = 0.004 (2‐tailed). This result survived FDR correction. For males 6 months of age, and males and females 10 months of age, barbering status was unable to predict the outcome of the stranger encounter tube test. No barbering was observed at 2 months. We concluded barbering status did not adequately predict the outcome of a stranger encounter in the Lindzey tube test, and excluded it from our subsequent analyses.

### 3.2 *Grb10* ^+/p^ barbers were no more common than WTs

**Figure 2:**
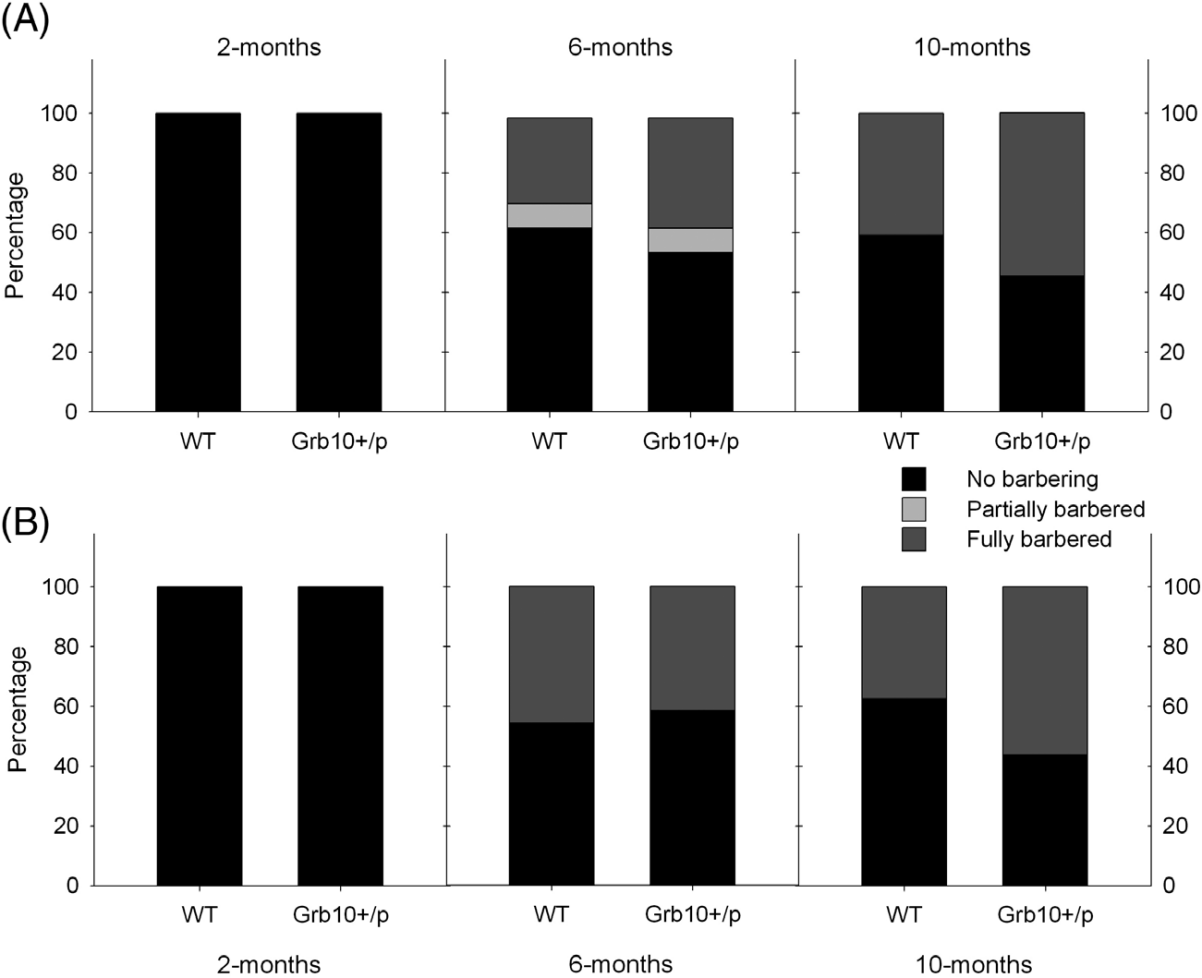
Whisker barbering in *Grb10^+/p^* socially house mice. Proportions of whisker barbering subdivided by genotype in A, Male and B, Female behavioral cohorts. Barbering was not present at 2 months, but tended to increase with age

Garfield 2011 reported an increased incidence of barbering in cages with *Grb10* ^*+/p*^ mice. In our study, behavioral cages at 6 and 10 months with identifiable barbers (1 un‐barbered to 3 barbered mice in the cage) were pooled to analyze the proportion of *Grb10* ^*+/p*^ vs WT barbers (Supplementary information, Table S2). Binomial tests indicated the proportion of barbers who were *Grb10* ^*+/p*^ was not statistically different from chance (0.5) in cages of either sex (Figure 2A,B; males *P* = 0.180, females *P* = 0.774, two‐tailed). After 30 days of isolation, none of the mice showed signs of whisker barbering.

### 3.3 Socially housed *Grb10* ^+/p^ mice do not show a social dominance phenotype

**Figure 3:**
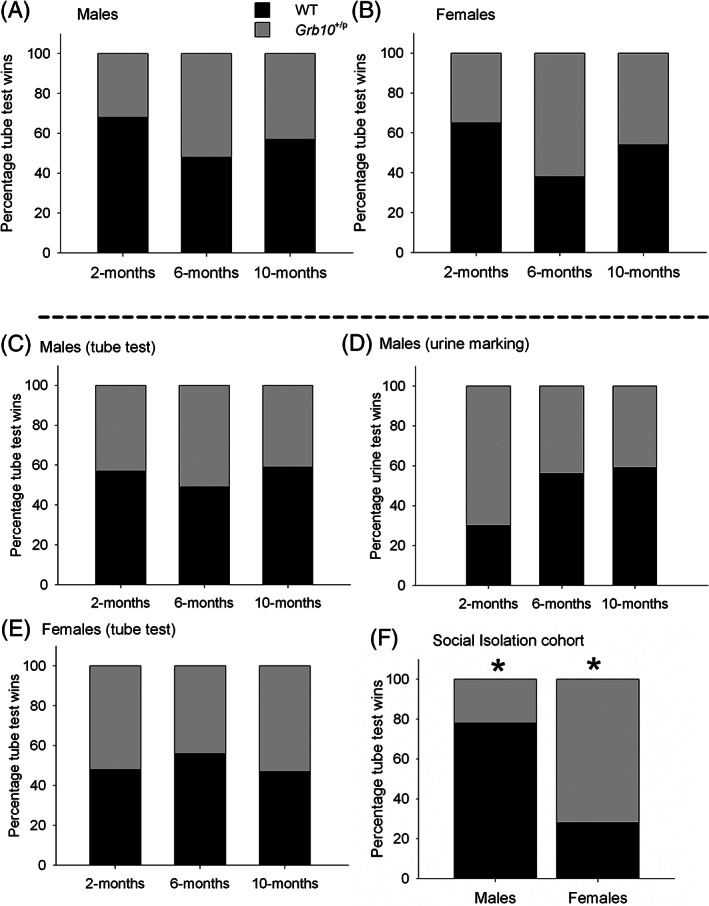
Social dominance tests in *Grb10^+/p^* mice. A, Male tube test wins vs strangers. B, Female tube test wins vs strangers. C, Male tube test wins vs cage mates. D, Male urine test wins vs cage mates. E, Female tube test wins vs cage mates. There were no significant genotype differences for any dominance tests conducted with socially housed mice. F, Male and female tube test wins vs strangers following 30 days of social isolation

In the stranger‐encounter (Figure 3A,B; Supplementary information, Table S3) and social encounter tube tests (Figure 3C,E; Supplementary information, Table S4 & S5), binomial analysis indicated the proportion of wins for Grb10^+/p^ mice in all three age groups for both sexes were not significantly different to chance (0.5). Likewise, the proportion of *Grb10* ^*+/p*^ wins in the urine marking test was not statistically higher than chance in the 6‐ and 10‐month cohorts. In the 2‐month cohort, the proportion of *Grb10* ^*+/p*^ wins in the urine marking test (0.70) at 2 months of age was statistically higher than chance (0.05), *P* = 0.01 (2‐tailed), but this did not survive FDR corrections (Figure 3D; Supplementary information, Table S6).

Rank hierarchies were also established in each cage using the social encounter tube and the urine marking tests (See Supplementary Figure S1). In the social tube test, there was no statistically significant difference between average within‐cage rank for *Grb10* ^*+/p*^ and WTs at 2, 6 or 10 months of age for males or females. In the urine marking test, there was no significant difference in average within‐cage rank at 6 and 10 months. At 2 months, the difference in urine marking rank between *Grb10* ^*+/p*^ mice (median average cage rank 0.667) and WTs (median average cage rank 0.333) was statistically significant, but this did not survive FDR correction. In both Garfield's testing (light/dark box, open field) and our own (elevated plus maze, see Supplementary Methods, Results and Figure S2), *Grb10* ^*+/p*^ mice did not display anxiety phenotypes which might confound social dominance testing.^3^, ^7^, ^19^, ^20^

### 3.4 Social isolation induces inconsistent effects on *Grb10* ^+/p^ dominance behavior

We replicated the social dominance paradigm in Garfield et al^3^ to determine if isolation stress was required to precipitate a social dominance phenotype. Naïve isolated *Grb10* ^*+/p*^ mice faced one naïve unfamiliar isolated WT per day for 3 days.^3^, ^7^ On Day 1, binomial analysis determined the proportions of male and female *Grb10* ^*+/p*^ wins were not statistically significantly different to chance (0.5). Over 3 days of stranger encounter tube tests, the proportion of male *Grb10* ^*+/p*^ wins (0.22) was statistically significantly lower than chance (0.50), *P* = 0.006 (2‐tailed), n = 27 matches. Conversely, the proportion of *Grb10* ^*+/p*^ female wins (0.72) over 3 days of stranger encounter tube tests was significantly higher than chance (0.5), *P* = 0.009 (2‐tailed), n = 39 matches (Figure 3F). Both significant results for male and female *Grb10* ^*+/p*^ wins over 3 days survived FDR corrections. Finally, the observed proportion of wins (0.69) for isolated female mice in oestrus, irrespective of genotype, was not statistically different to chance (0.5), *P* = 0.267 (2‐tailed), n = 13 matches.

### 3.5 Socially housed mixed genotype cages show signs of social hierarchy instability

**Figure 4:**
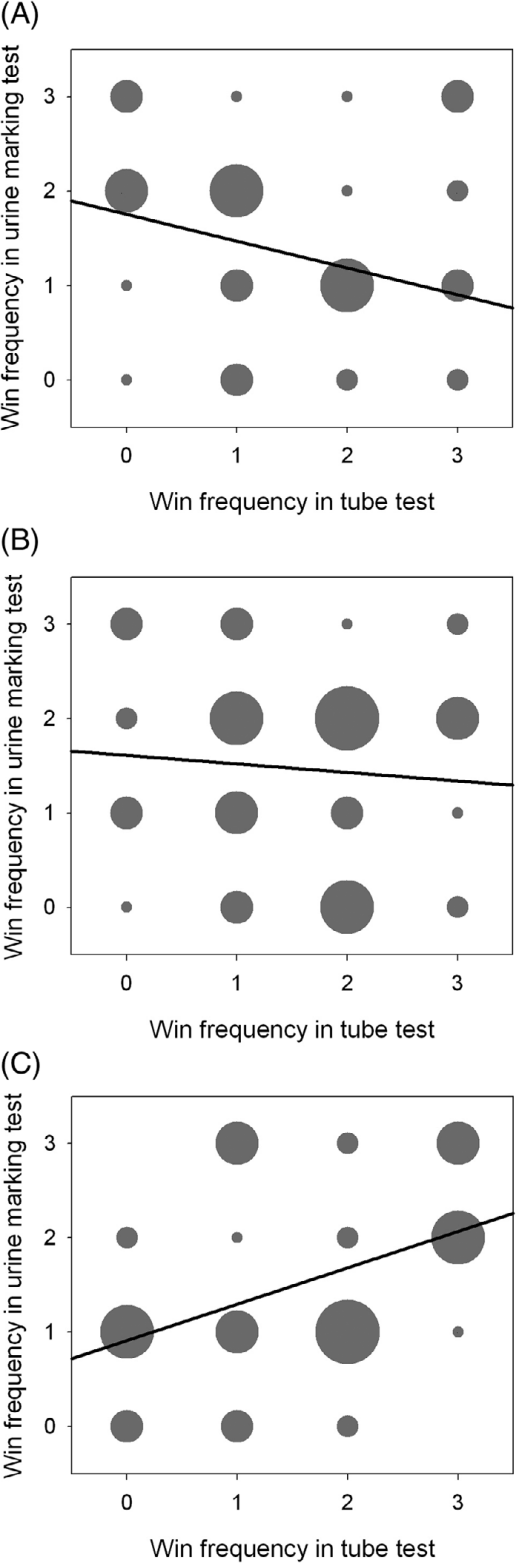
No correlation between social dominance measures in mixed cages of *Grb10^+/p^* and WT mice. Win frequency (0, 1, 2 or 3 wins) in the urine marking test was plotted against frequency in the social tube test for each male mouse. A, Males 2 months; B, Males 6 months; C, Males 10 months. There was initially a significant linear association at 10 months, but this did not survive FDR correction

While male cohorts had a higher absolute proportion of linear hierarchies in the social dominance tests than females (Supplementary Figure S1), both sexes showed evidence of transitivity within each test. Consequently, cage ranks determined by the social tube test, urine marking test and barbering status were analyzed for linear correlation. Different tests of social dominance are expected to correlate,^21^ and indeed we have previously seen this in our lab.^22^ However, there was no significant linear association between rank in the social tube test and rank in the urine marking test for the male behavioral cohorts 2 and 6 months of age (Figure 4). At 10 months of age there was a significant linear association between tube test rank and urine marking rank, χ^2^(1) = 7.176, *P* = 0.007, *r* = 0.409, n = 44. When this cohort was broken down by genotype group, a significant linear association was found for male *Grb10* ^*+/p*^ (χ^2^(1) = 5.706, *P* = 0.017, *r* = 0.521) mice, but not for WTs.

**Figure 5:**
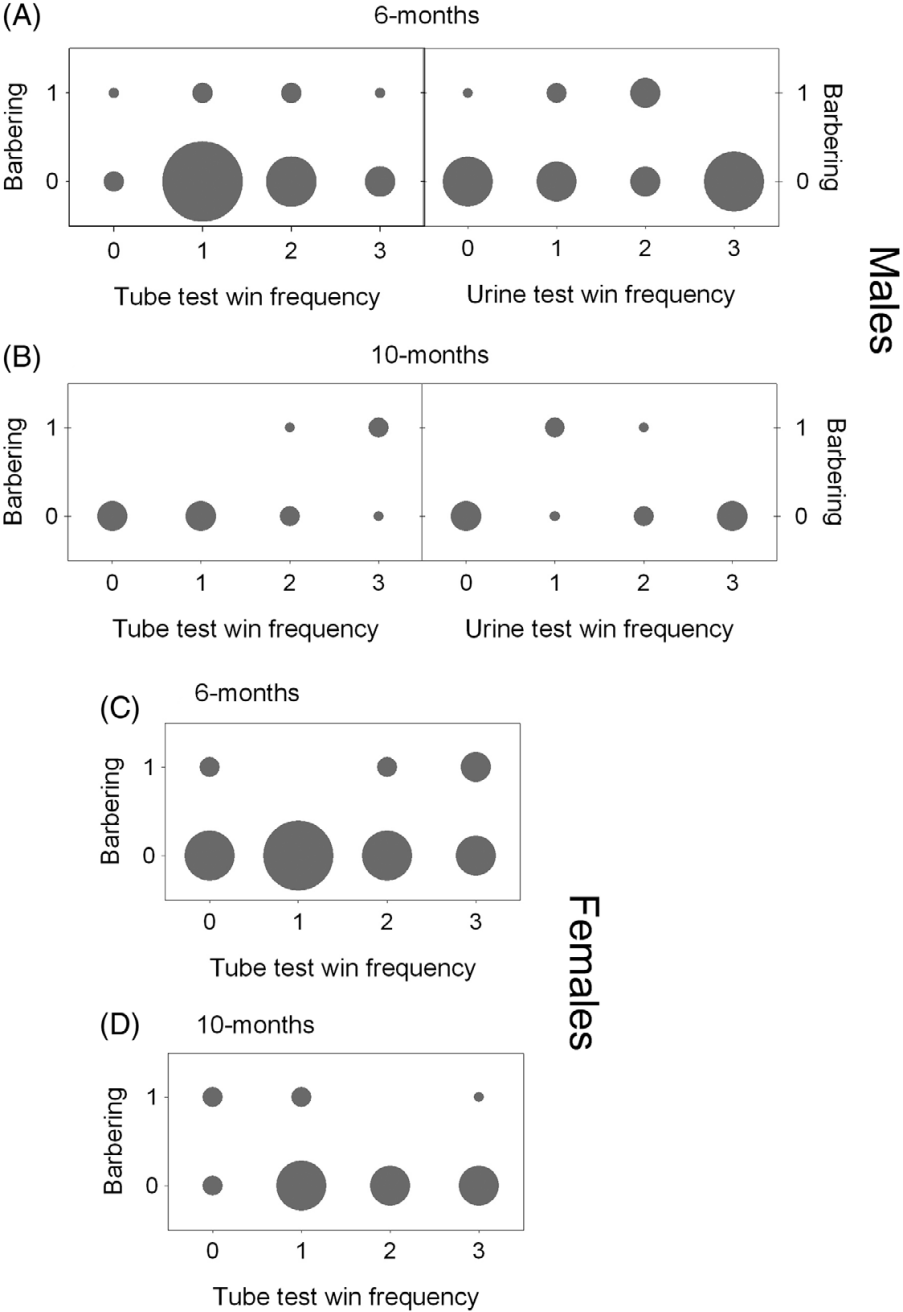
No correlation between social dominance measures and barbering in mixed cages of *Grb10^+/p^* and WT mice. Barbering status (0 –subordinate barbered mouse, 1 –dominant barber) was plotted against win frequencies in the social tube and urine tests (0, 1, 2 or 3 wins). Barbering plotted for A, male mice at 6 months against social tube and urine tests; B, male mice at 10 months against social tube and urine tests; C, female mice at 6 months against social tube test and D, female mice at 10 months against social tube test. There was no barbering at 2 months (See Figure 1)

Additionally, there was a significant linear association between tube test and barbering rank for male mice (pooled genotypes) 10 months of age (χ^2^(1) = 3.993, *P* = 0.046, *r* = 0.602, n = 12) (Figure 5). When the cohort was broken down by genotype group, male WTs (χ^2^(1) = 4.091, *P* = 0.043, *r* = 0.905) but not *Grb10* ^*+/p*^ mice had a linear association. All other associations between barbering and social tube (male and female) or barbering and urine ranking (male) mice were not significant (Figure 4). Although the four associations of cage rank above were originally found to be significant, none survived FDR correction.

## 4 DISCUSSION

Our primary goal was to assess social dominance behavior in group‐housed *Grb10* ^*+/p*^ mice at multiple ages. Social housing provided a more ecologically relevant context for social dominance strategies optimal in close quarters. Group housed animals benefit from social hierarchies reducing costly conflicts, in contrast to isolation housing, where more territorial and aggressive confrontation strategies are more beneficial.^10^, ^23^ We examined three cohorts, at 2, 6 and 10 months of age, to capture any variation in dominance or hierarchical behaviors that might depend on age. Barbering, for instance, was absent in our 2‐month cohorts, and appeared in cohorts 6 and 10 months of age. Male and female mice underwent testing to determine whether sex‐specific strategies were differentially impacted by paternal *Grb10* deletion.^18^

In both sexes and all three age groups, we found no difference between *Grb10* ^*+/p*^ and WT socially housed mice in likelihood of winning matches in the stranger‐encounter Lindzey tube test, familiar‐encounter Lindzey tube test or urine marking test. In the two within‐cage dominance tests, we found no significant genotype differences in average cage rank. Additionally, the proportion of *Grb10* ^*+/p*^ barbers pooled across all three age groups was not statistically significantly different from chance. From this convergent evidence across large cohorts of both sexes at multiple ages, we concluded socially housed *Grb10* ^*+/p*^ mice do not show enhanced social dominance.

These results contrasted the previously reported enhanced dominance phenotype of isolated *Grb10* ^*+/p*^ male mice in tube test matches against unfamiliar mice.^3^ We next replicated the conditions of the Garfield 2011 study to assess whether social isolation stress precipitated the social dominance phenotype in *Grb10* ^*+/p*^ mice. In our isolation studies, *Grb10* ^*+/p*^ males were statistically significantly *less* likely to win in the stranger‐encounter Lindzey tube test against an unfamiliar socially isolated WT opponent. This result was opposite to the finding reported in reference 3. Although these experiments were run in different labs (Bath and Cardiff), we replicated the background strain (the mice were derived from the original Bath colony), the conditions of testing and the power of the experiment.^3^, ^7^ Notably, we chose not to use a statistical re‐sampling technique such as the Monte Carlo permutation test, because of concerns about amplifying noise.^3^ In contrast to males, our *Grb10* ^*+/p*^ females were statistically significantly *more* likely to win in the stranger‐encounter Lindzey tube test.

Our data suggest sex‐specific effects of isolation on social dominance behaviors in our *Grb10* ^*+/p*^ mice. Sex differences in the expression of (presumably) maternal *Grb10* in muscle have been noted,^24^ but as far as we are aware there are no known sex‐differences in terms of paternal *Grb10* expression in the brain,^25^ although this has yet to be explored systematically. However, taken together with the Garfield study, the opposing direction of effects in male *Grb10* ^*+/p*^ mice following isolation do not suggest enhanced social dominance is necessarily a consistent consequence of social isolation. Rather that there is an interaction between *Grb10* expression and isolation that produces a change in social dominance related behaviors. This may be mediated via altered monoaminergic signaling in the midbrain.^11^, ^12^ For instance, *Grb10* ^*+/p*^ mice lack normal expression in dopamine neurons of the dorsal raphe nucleus.^3^ This population represents the experience of social isolation, and this experience is modulated by an individual's prior social rank.^14^ *Grb10* ^*+/p*^ mice possibly experience social isolation stress differently, or employ altered social strategies in hierarchical conflicts following isolation stress.^14^, ^23^

Agreement between dominance tests is important in showing a given test measures social dominance as an underlying dependent variable, rather than measuring differences in the sensorimotor skills required to undertake the test. Convergent tests strengthen the description of a robust dominance hierarchy and the characterization of a social dominance phenotype.^10^, ^21^ We found both *Grb10* ^*+/p*^ male and female cages formed linear hierarchies. We therefore performed tests of rank association between our social tube, urine marking and barbering data. While four associations were originally significant, none remained so after FDR correction. However, reports of barbering and tube test rank correlations in the literature suggest the use of training prior to the tube test results in correlation between these dominance measures, whereas the absence of training does not result in correlation.^10^ To match the protocols reported in reference 3 and to avoid learning effects, we did not use tube test training, and this may be relevant to interpreting the absence of correlation between barbering and tube test results. Regardless, we note successful correlation between tube test (without training) and urine marking ranks in unrelated control colonies.^22^

A comparable phenotype, interpreted as social instability, is present in the *Cdkn1c* ^*BACx1*^ mouse model, which overexpresses imprinted cyclin dependent kinase inhibitor 1c (*Cdkn1c*).^22^ Social instability has adverse effects on individual fitness including anxiety, stress and reduced breeding rates.^26^, ^27^, ^28^ *Cdkn1c* ^*BACx1*^ mice do not occupy more dominant ranks than their WT cage‐mates on any individual measure of within‐cage social hierarchy. However, in *Cdkn1c* ^*BACx1*^ containing cages, an individual's rank in one dominance measure did not correlate with its rank in another.^22^ Clear transitive hierarchies in individual measures of social dominance form in both *Cdkn1c* ^*BACx1*^/WT and *Grb10* ^*+/p*^/WT cages, but these are demonstrably unstable in *Cdkn1c* ^*BACx1*^ colonies.^22^ Nevertheless, a different experimental set up is required to determine within‐cage rank stability over time for social groups with *Grb10* ^*+/p*^ animals. It is also possible *Grb10* ^*+/p*^ mice alter the behavior of WT littermates, as is the case for *Cdkn1c* ^*BACx1*^ and *Nlgn3*.^22^, ^29^ Our *Grb10* ^*+/p*^ and WT balanced cage set up lacks an appropriate independent control group, like cages of *Cdkn1c* ^*BAClacZ*^ and WT mice,^22^ to test this.

We have showed through robust and convergent testing at multiple ages, and in both sexes, that socially housed *Grb10* ^*+/p*^ mice do not show a social dominance phenotype. Nevertheless, following social isolation there is an interaction with *Grb10* expression that produces a change in social dominance related behaviors, with a sexually dimorphic direction of effects; critically the direction of effects was contrary to previous findings.^3^ We also noted an absence of correlation of hierarchical rank between different dominance tests undertaken by *Grb10* ^*+/p*^ containing cages, a pattern of behavior previously proposed to indicate instability of social rank.^22^ Taken together, these findings suggest that paternal *Grb10* may influence stability of social behavior. Nevertheless, although it is clear from the work here and others^30^ that paternal *Grb10* does impact on brain function generally, further work is required to determine the exact role played in social behavior.

## CONFLICT OF INTEREST

The authors declare they have no competing interests.

## Supporting information


**Data S1.**
