# Supplementary material for: Detailed analysis of paternal knockout Grb10 mice suggests effects on stability of social behavior, rather than social dominance
Source: Genes Brain Behav. 2019 Apr 29;19(1):e12571. doi: 10.1111/gbb.12571 (PMC7050506; doi:10.1111/gbb.12571)
Supplement: Supplementary file 1 — Data S1. [file GBB-19-e12571-s001.docx]

**Detailed analysis of paternal knockout *Grb10* mice suggests effects on stability of social behavior, rather than social dominance**

**– *Supplementary information***

Kira D. A. Rienecker^1^, Alexander T. Chavasse^1^, Kim Moorwood^2^, Andrew Ward^2^ & Anthony R. Isles^1^*

^1^MRC Centre for Neuropsychiatric Genetics and Genomics, Neuroscience and Mental Health Research Institute, School of Medicine, Cardiff University, Hadyn Ellis Building, Maindy Road, Cardiff, CF24 4HQ, UK

^2^Department of Biology and Biochemistry, University of Bath, Building 4 South, Bath, BA2 7AY, UK

*Correspondence: Anthony R. Isles, IslesAR1@cardiff.ac.uk

**SUPPLEMENTARY METHODS**

| Table S1 Stranger Encounter Tube Test–Trials Not Counted Due to Failure Total encounters attempted and failed in the stranger encounter tube test. Only successful trials were included in the analysis. | | | | |
| --- | --- | --- | --- | --- |
| Totals | F Attempted | F Failed | M attempted | M Failed |
| Cohort D | 24 | 0 | 28 | 0 |
| Cohort C | 24 | 3 | 26 | 3 |
| Cohort A/B | 16 | 3 | 26 | 0 |
| Isolation Day 1 | 15 | 4 | 10 | 2 |
| Isolation Day 2 | 15 | 2 | 10 | 1 |
| Isolation Day 3 | 15 | 0 | 10 | 0 |

**Elevated Plus Maze (EPM)**

The Elevated Plus Maze was carried out in a quiet room with overhead fluorescent lighting, which was necessary for Ethovision detection. The maze consisted of two bisecting white arms 80mm in width by 430mm in length and was elevated 45 cm above the foundation. The opposing pairs of arms were designated “Closed arms” (with 17cm high walls) and “Open arms” (without walls) respectively. The center square of 80mm x 80mm was designated “Middle”. One cage of four mice was carried into the testing room at a time, and remained until all cage mates had individually completed the task. To begin the 5-minute trial, mice were placed in Closed Arm 1. Movement was recorded by the Ethovision detection system, while time for grooming, stretch-attend, and head dips over the edge were scored manually. Between trials, the maze was cleaned with 70% alcohol wipes. Data for Ethovision measures in the EPM task were analyzed using a two-way ANOVA, with AGE and GENOTYPE as between-subjects independent variables, and an Ethovision measure as the dependent variable. Data in main effects analyses are presented as estimated marginal mean ± standard error of the estimated marginal mean, unless otherwise stated. Graphs report descriptive means ± standard error of the descriptive mean, unless otherwise stated. One-way ANOVA was used for each age bin separately when two-way ANOVA was not possible.

**SUPPLEMENTARY RESULTS**

| *Table S2 Barber Genotype in Behavioral Cohorts*  Cages with a clear 1:3 dominant barber to subordinate barbered mouse ratio were pooled across age for analysis. There was no statistically significant difference in genotype frequency among barbers. | | | | |
| --- | --- | --- | --- | --- |
| Sex | Barbered Cages  (pooled) | WT Barbers | *Grb10^+/p^* Barbers | Sig. |
| Male | 9 cages | 0.78 | 0.22 | 0.180 |
| Female | 12 cages | 0.58 | 0.42 | 0.774 |

| ***Table S3 Stranger Tube Test Grb10^+/p^ Proportions and P-values***  Statistical analysis of socially housed *Grb10^+/p^* proportion wins against unfamiliar wildtypes in the stranger encounter tube tests. | | | | | | |
| --- | --- | --- | --- | --- | --- | --- |
| **Age** | **Male matches (N)** | **Males proportion wins** | **Males**  **p value** | **Female matches (N)** | **Females proportion wins** | **Females**  **p value** |
| 2 months | 28 | 0.320 | 0.087 | 20 | 0.350 | 0.263 |
| 6 months | 23 | 0.522 | 1.000 | 21 | 0.619 | 0.383 |
| 10 months | 23 | 0.430 | 0.678 | 13 | 0.462 | 1.000 |

| *Table S4 Males Social Tube Test Grb10^+/p^* *vs WT Binomial Analysis*  Statistical analysis of socially housed male *Grb10^+/p^* proportion wins in matches against wildtype cage mates in the social tube test and proportion of linear hierarchies in genotype balanced cages completing the social tube test. | | | | |
| --- | --- | --- | --- | --- |
| Age | Male matches (N) | Males proportion wins Grb10 +/p | Males  p value | Linear Hierarchy |
| 2 months | 56 | 0.43 | 0.350 | 11/12 |
| 6 months | 51 | 0.51 | 1.000 | 8/12 |
| 10 months | 46 | 0.41 | 0.302 | 9/11 |

| *Table S5 Females Social Tube Test Grb10^+/p^ vs WT Binomial Analysis*  Statistical analysis of socially housed female *Grb10^+/p^* proportion wins in matches against wildtype cage mates in the social tube test and proportion of linear hierarchies in genotype balanced cages completing the social tube test. | | | | |
| --- | --- | --- | --- | --- |
| Age | Female matches (N) | Females proportion wins Grb10 +/p | Females  p value | Linear Hierarchy |
| 2 months | 40 | 0.53 | 0.875 | 5/10 |
| 6 months | 48 | 0.44 | 0.471 | 10/12 |
| 10 months | 32 | 0.53 | 0.860 | 4/8 |

| *Table S6 Males Urine Marking Test Grb10^+/p^ vs WT Binomial Analysis*  Statistical analysis of socially housed male *Grb10^+/p^* proportion wins in matches against wildtype cage mates in the urine marking test and proportion of linear hierarchies in genotype balanced cages completing the urine marking test. A significant difference at 2 months did not survive FDR correction. | | | | |
| --- | --- | --- | --- | --- |
| Age | Male matches (N) | Males proportion wins Grb10 +/p | Males  p value | Linear Hierarchy |
| 2 months | 44 | 0.70 | 0.01*did not survive FDR | 8/11 |
| 6 months | 52 | 0.56 | 0.488 | 9/12 |
| 10 months | 46 | 0.41 | 0.302 | 8/11 |

| \| **A**  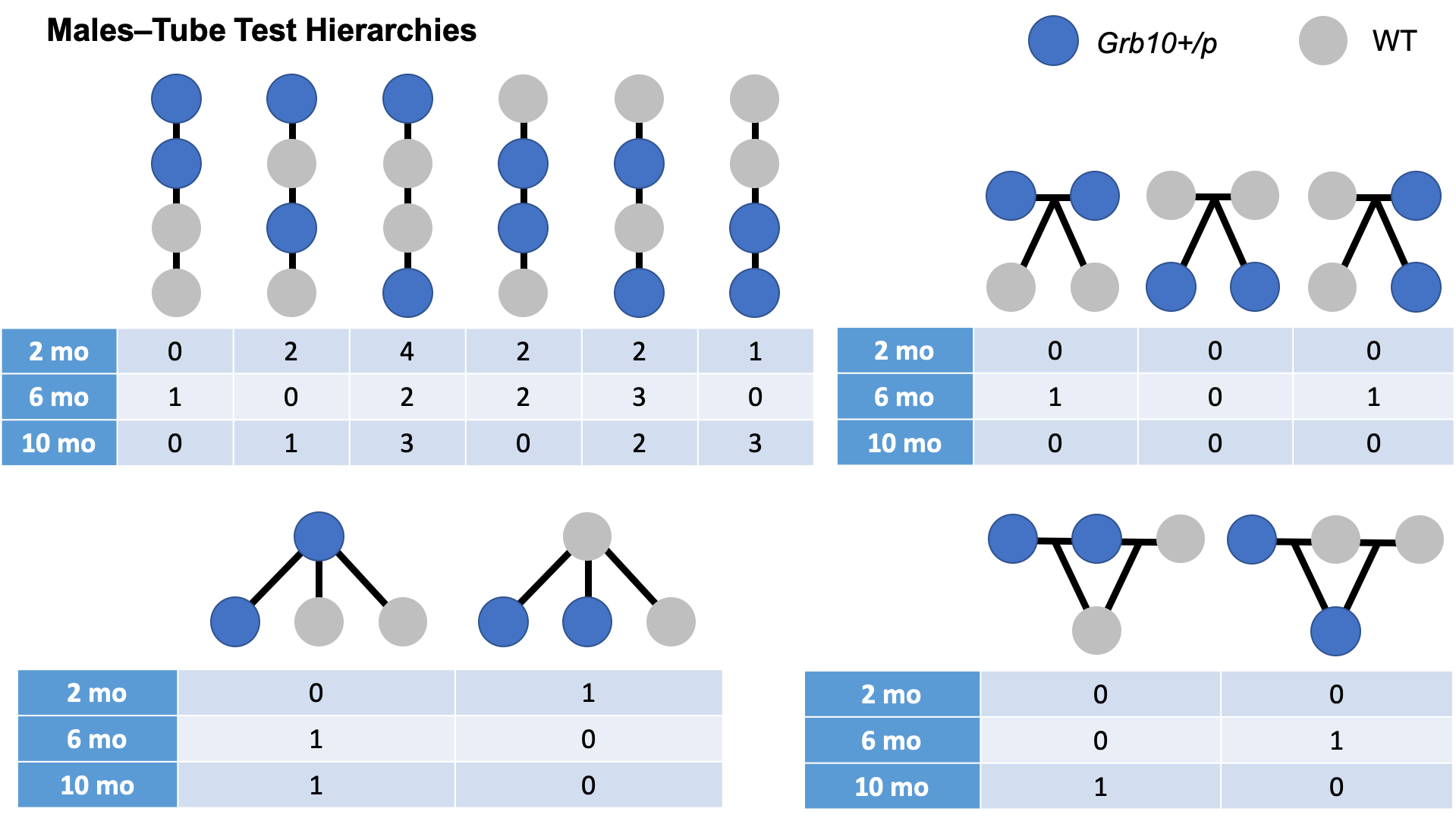 \| \| --- \| \| **B**  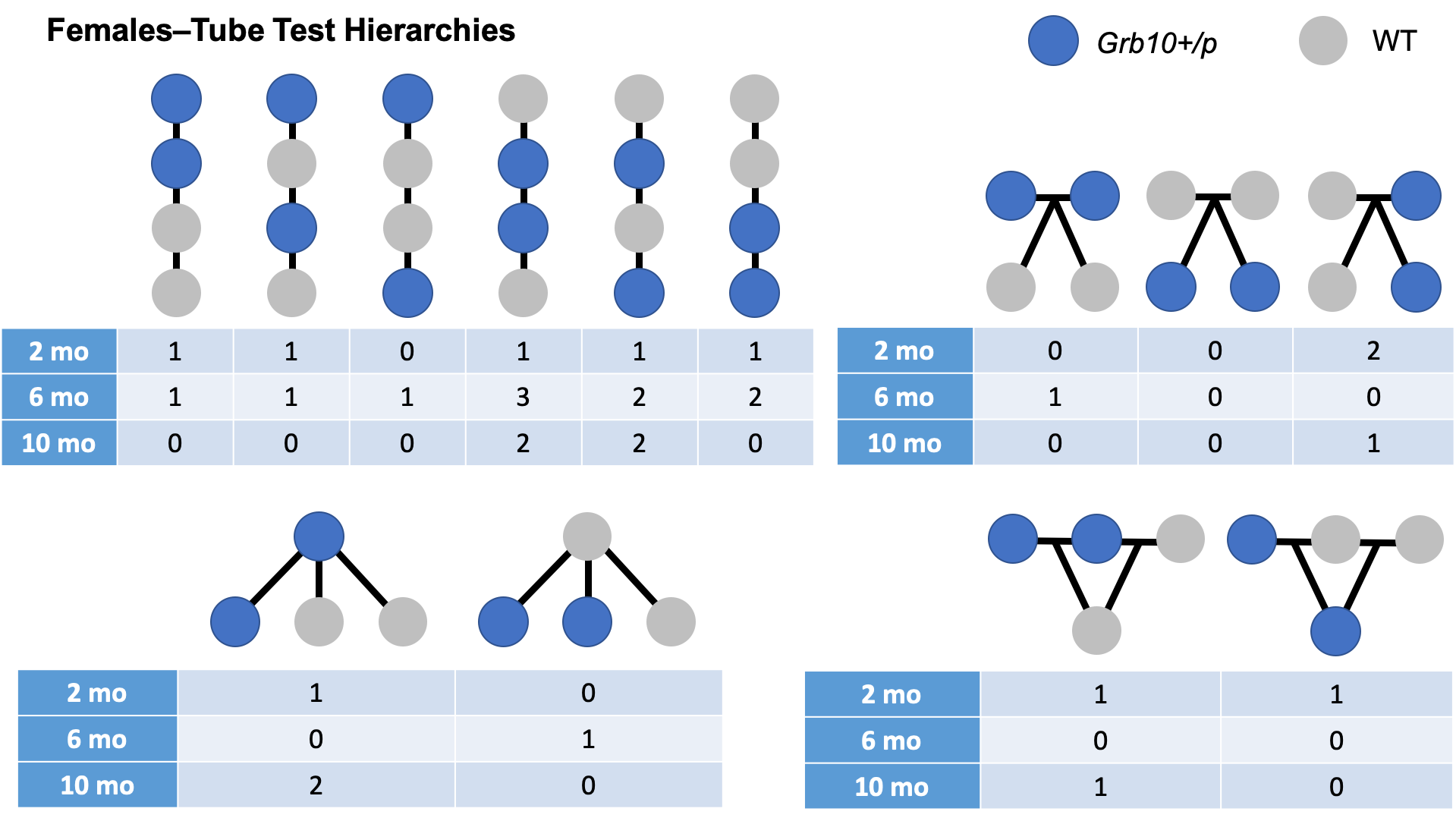 \| \| **C**  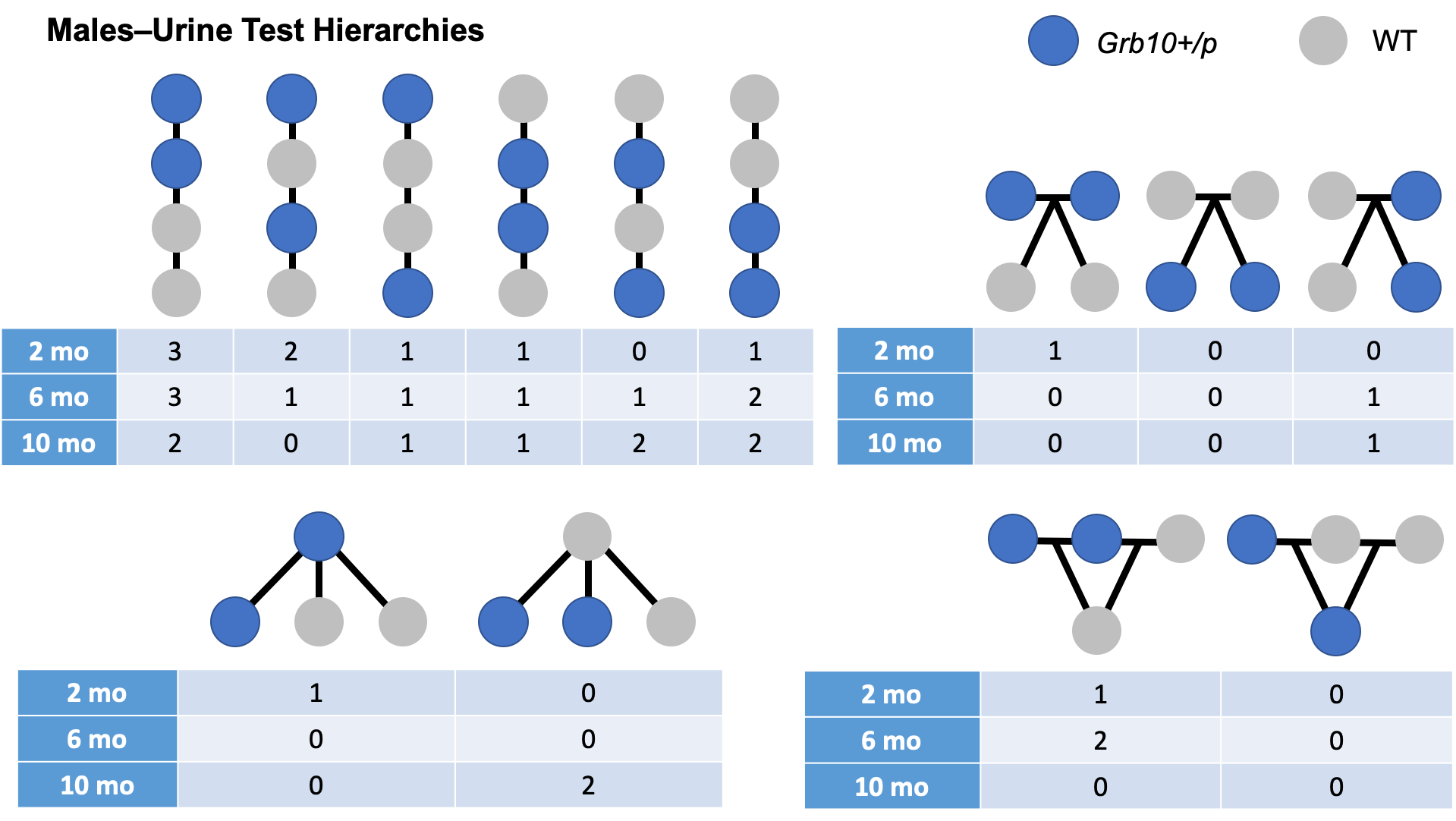 \|   **Figure S1 Social dominance hierarchy structures in *Grb10*^+/p^ and WT social groups**  All possible hierarchies for 4-mouse cages are depicted with blue (*Grb10^+/p^*) and grey (WT) circles connected by lines representing dominance relationships based on number of wins. The most dominant mice (higher number of wins) are towards the top of each diagram. Frequencies of each hierarchy within each cohort are displayed below the diagrams. A) Male social tube test hierarchies, B) Female social tube test hierarchies, C) Male urine marking test hierarchies. |
| --- | --- | --- | --- |

**Body Weight Analysis**

A three-way ANOVA was conducted to examine the effects of between-subjects factors GENOTYPE, AGE, and SEX on body weight in our colony and behavioral groups. GENOTYPE was considered at three levels (wildtype, *Grb10^+m^*, and *Grb10^+/p^*), AGE was considered at three levels (75-95 days, 185-205 days, and 305-325 days of age), and SEX was considered at two levels (Females and Males). The three-way interaction between GENOTYPE, SEX, and AGE was not statistically significant, nor was the two-way interaction between SEX and AGE. The two-way interactions between GENOTYPE*SEX (F(2,276) = 3.134, p = 0.045, partial η^2^ = 0.022) and AGE*GENOTYPE (F(4,276) = 4.141, p = 0.003, partial η^2^ = 0.057) were statistically significant, and we followed up with simple main effects analysis. For GENOTYPE*SEX, the simple main effect of GENOTYPE was not significant for male or female mice separately, but there was a significant main effect of SEX for wildtype, *Grb10^+/m^*, and *Grb10^+/p^* mice individually. Male body weights were consistently heavier than female body weights for all three genotype groups. For AGE*GENOTYPE, the simple main effect of AGE was significant for each genotype group individually. Wildtype and *Grb10^+/p^* mice weighed significantly more at each consecutive age group (75-95 days < 185-205 days < 305-325 days), while *Grb10^+/m^* mice weighed more at 305-325 days than at 185-205 and 75-95 days, but were not significantly different between 75-95 days and 185-205 days. The simple main effect of GENOTYPE for AGE*GENOTYPE was significant for 305-325 days and 75-95 days, but not 185-205 days of age. No pairwise comparisons between the genotype groups at 305-325 days survived Bonferroni correction. At 75-95 days, *Grb10^+/m^* mice were significantly heavier than both wildtype and *Grb10^+/p^* mice, and there was no significant difference between wildtype and *Grb10^+/p^* body weights.

**Elevated Plus Maze (EPM)**

***Entries to Open Arm***

At 10 weeks, total “open arm entries” was not statistically significantly different between *Grb10^+/p^* (19.478 ± 6.626 entries) and wildtype (16.783 ± 7.722 entries) mice, F(1,44) = 1.614, p = 0.211, partial η^2^ = 0.035. At 6 months, “open arm entries” were statistically different between *Grb10^+/p^* (15.700 ± 6.182 entries) and wildtype (9.955 ± 6.484) trials F(1,40) = 8.596, p = 0.006, partial η^2^ = 0.177. This did not survive FDR correction. At 10 months, the assumption of homogeneity of variance was violated (Levene’s test p = 0.019). Therefore, we interpreted Welch’s ANOVA. There was no statistically significant difference in “open arm entries” between *Grb10^+/p^* (16.286 ± 12.546 entries) and wildtype (11.000 ± 6.347 entries) trials, Welch’s F(1,29.300) = 2.995, p = 0.094.

***Total Entries***

As there was a significant genotype difference in total open arm entries at 6 months of age (pre-FDR correction), we also examined total entries to all zones of the EPM to determine if this effect was specific to the open arm. The interaction between GENOTYPE and AGE was not statistically significant for “all entries”, F(2,125) = 0.631, p = 0.534, partial η^2^ = 0.010. Therefore, analyses for main effects were performed. There was a statistically significant main effect of GENOTYPE for “all entries”, F(1,125) = 17.909, p < 0.001, partial η^2^ = 0.125. This survived FDR correction. *Grb10^+/p^* mice made more entries to EPM zones (82.834 ± 2.898 entries) than wildtype mice (65.698 ± 2.828 entries), mean difference 17.137 (95%CI 9.122 to 25.151) entries, p < 0.001.

There was a statistically significant main effect of AGE on “all entries”, F(2,125) = 6.709, p = 0.002, partial η^2^ = 0.097. Mice at 10 weeks made the most entries (84.565 ± 3.413 entries), while mice at 6 months (68.155 ± 3.575 entries) and 10 months (70.078 ± 3.531 entries) made fewer. Mice 10 weeks of age made significantly more entries than mice at 6 months, mean difference 16.411 (95% CI 4.417 to 28.404) entries, p = 0.004. Mice 10 weeks of age also made 14.487 (95%CI 2.572 to 26.402) entries than mice at 10 months, p = 0.011. There was no statistically significant difference between “all entries” made by mice at 6 months and 10 months, mean difference -1.923 (95%CI -14.117 to 10.270) entries, p = 1.000. The main effect of AGE and the pairwise comparisons did not survive FDR correction. Overall, there was a significant genotype difference in “all entries” made to zones of the EPM, indicating increased entries by *Grb10^+/p^* mice at 6 months was not specific to the open arm.

***Percent Time in Open Arms***

As the increase in entries made by *Grb10^+/p^* mice was not specific to the open arm, we examined the division of time to determine whether *Grb10^+/p^* mice differed in the amount of time spent on the open arm. There was no statistically significant interaction between GENOTYPE and AGE for “percent time in open arms”, F(2,125) = 1.226, p = 0.297, partial η^2^ = 0.019. Therefore, analyses for main effects were performed. There was a statistically significant main effect of GENOTYPE on “percent time in open arms”, F(1,125) = 7.727, p = 0.006, partial η^2^ = 0.058. *Grb10^+/p^* mice spent significantly more time on the open arm (19.094 ± 1.390%) than wildtypes (13.697 ± 1.356%), mean difference 5.398 (95%CI 1.555 to 9.241) %, p = 0.006. This effect of GENOTYPE did not survive FDR correction.

There was a statistically significant main effect of AGE on “percent time in open arms”, F(2,125) = 5.786, p = 0.004, partial η^2^ = 0.085. Mice 10 weeks of age spent 20.823 ± 1.636%, 6 months of age spent 15.289 ± 1.715%, and 10 months of age spent 13.074 ± 1.693% of the total time on open arms. Time at 10 weeks was statistically higher than at 10 months (7.749 (95%CI 2.035 to 13.462) %, p = 0.004, but not than at 6 months (5.534 (95%CI -0.217 to 11.285) %, p = 0.063. There was no significant difference between percent time spent on open arms at 6 months and 10 months (2.214 (95%CI -3.633 to 8.062) %, p = 1.000. Neither the main effect of AGE, nor the pairwise comparisons survived FDR correction.

*Grb10^+/p^* mice make more total entries to EPM zones than wildtypes, but do not make more entries to the open arm, nor spend more time on the open arm, when analyses are adjusted for FDR.

| **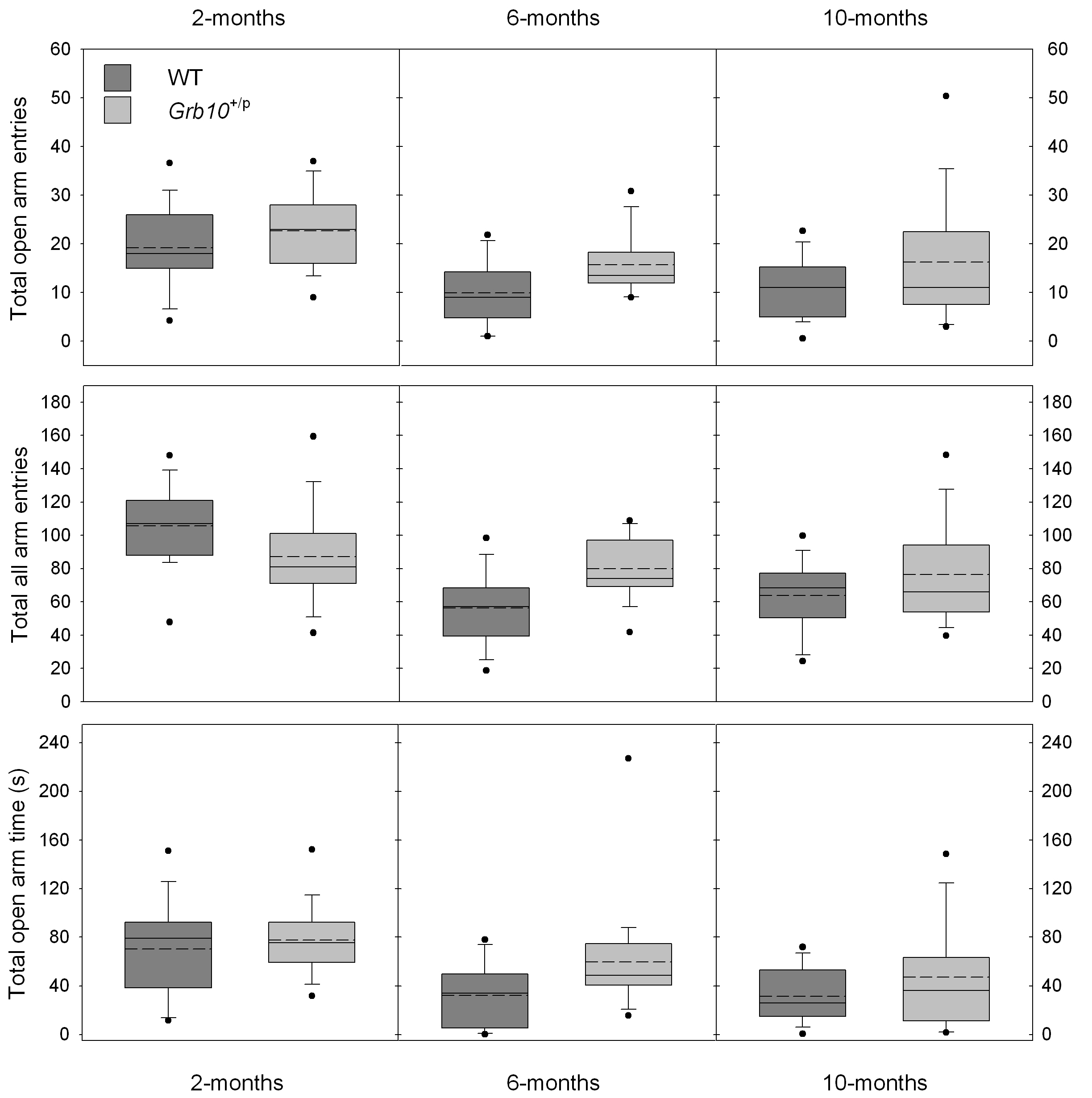**  **Figure S2 Behavior of *Grb10*^+/p^ mice in the elevated plus maze**.  Performance in the EPM for 2 month, 6 month, and 10 month male behavioral cohorts. Male *Grb10^+/p^* mice made significantly more total all arm entries, but did not make more total open arm entries (after FDR correction) or spend more total time on the open arm. Data are box-plots showing median (solid line), mean (dashed line) and 5^th^, 25^th^ and 75^th^ and 95^th^ percentiles |
| --- |
